# Role of TREK-1 in Health and Disease, Focus on the Central Nervous System

**DOI:** 10.3389/fphar.2019.00379

**Published:** 2019-04-11

**Authors:** Alaeddine Djillani, Jean Mazella, Catherine Heurteaux, Marc Borsotto

**Affiliations:** Centre National de la Recherche Scientifique, Institut de Pharmacologie Moléculaire et Cellulaire, UMR7275, Université Côte d’Azur, Valbonne, France

**Keywords:** TREK-1, potassium, ion channel, K_2P_, modulators, neurological disorders, arrhythmia

## Abstract

TREK-1 is the most studied background K_2P_ channel. Its main role is to control cell excitability and maintain the membrane potential below the threshold of depolarization. TREK-1 is multi-regulated by a variety of physical and chemical stimuli which makes it a very promising and challenging target in the treatment of several pathologies. It is mainly expressed in the brain but also in heart, smooth muscle cells, endocrine pancreas, and prostate. In the nervous system, TREK-1 is involved in many physiological and pathological processes such as depression, neuroprotection, pain, and anesthesia. These properties explain why many laboratories and pharmaceutical companies have been focusing their research on screening and developing highly efficient modulators of TREK-1 channels. In this review, we summarize the different roles of TREK-1 that have been investigated so far in attempt to characterize pharmacological tools and new molecules to modulate cellular functions controlled by TREK-1.

## Introduction

In the brain, ion channels regulate a variety of cellular processes such as neurotransmitter release, neuronal excitability, and plasticity. They are crucial in the generation and propagation of action potentials. Dysfunction of these channels causes several pathologies called channelopathies. In order to treat these channelopathies, many ion channels have been targets of small molecules and biological drugs. Overall, ion channels represent 19% of all human protein targets (Santos et al., 2017). To date, 177 ion channels-based drugs have been approved as treatments for various pathologies and disorders (Santos et al., 2017). Central nervous system (CNS) pathologies were associated with dysregulation of ion channels such as voltage gated Na^+^, K^+^, and Ca^2+^ channels. Calcium channels are the most studied among ion channels since Ca^2+^ ions in addition to controlling the cell excitability, they act as second messengers that convert electrical signals into chemical activity (Pietrobon, 2002). On the other hand, to control neuronal excitability, potassium channels repolarize neurons by extruding K^+^ ions to the extracellular space. Neurological potassium channelopathies are mainly caused by dysregulation of voltage-dependent potassium channels (Benatar, 2000) and also of the inwardly rectifying potassium channels (Kir) which provokes an hyperexcitability seen in epilepsy (Spillane et al., 2016). Mutations in the KCNT1 gene encoding for sodium -activated potassium channels cause a rare infantile encephalopathy called the migrating partial seizures of infancy (Barcia et al., 2012). In other cases, mutations of KCNQ2 encoding for Kv7.2 channel provoke severe epileptic encephalopathies (Weckhuysen et al., 2012). The inward-rectifier potassium channel Kir 6.2 is encoded by KCNJ11 gene forms the major subunit of the ATP-sensitive potassium channel. Mutations in this gene cause DEND syndrome, a very rare severe form of neonatal diabetes mellitus characterized by developmental delay, epilepsy and neonatal diabetes (Gloyn et al., 2004). Kv7.2 and Kv7.3 are the main components of the slow voltage-gated M-channel which regulates neuronal excitability (Brown and Passmore, 2009). Loss of function mutations of the KCNQ2 and KCNQ3 genes encoding, respectively, for Kv7.2 and Kv7.3 causes benign familial neonatal convulsions (Spillane et al., 2016). A dominant missense mutation in KCNMA1 gene encoding for pore-forming α-subunit of the large conductance calcium-sensitive potassium channel (BK), has been associated with a form of generalized epilepsy and paroxysmal dyskinesia (Du et al., 2005).

Some mutations in K_2P_ channels were reported to cause several pathologies. Birk Barel mental retardation dysmorphism syndrome is caused by a missense mutation in the maternal copy of KCNK9 gene which encodes for TASK-3 (K_2P_9.1). Loss of function of the channel was observed in homodimers and also when it forms heterodimer with TASK-1 (K_2P_.3.1) (Barel et al., 2008). Recently, *de novo* a gain of function missense mutation of KCNK4 gene encoding for TRAAK channel, was reported to cause recognizable neurodevelopmental syndrome characterized by a facial dysmorphism, hypertrichosis, epilepsy, intellectual disability/developmental delay and gingival overgrowth (Bauer et al., 2018). A frameshift mutation (F139Wfsx24) in KCNK18 encoding for the calcium-activated K_2P_ channel TRESK channel was associated with migraine with aura (Lafreniere et al., 2010). Channelopathies caused by dysfunction of potassium channels are of high interest for researchers since they may present interesting targets for potential treatments.

The most recent discovered family of two-pore domain potassium channels (K_2P_) are regulated by a variety of chemical and physical stimuli (Lesage and Lazdunski, 2000). In this review, we will focus on describing the role of one of the most studied K_2P_ channel, TREK-1 channel in health and disease. In addition to discussing the recent pharmacological modulators of its activity.

## Trek-1 Channel

**FIGURE 1:**
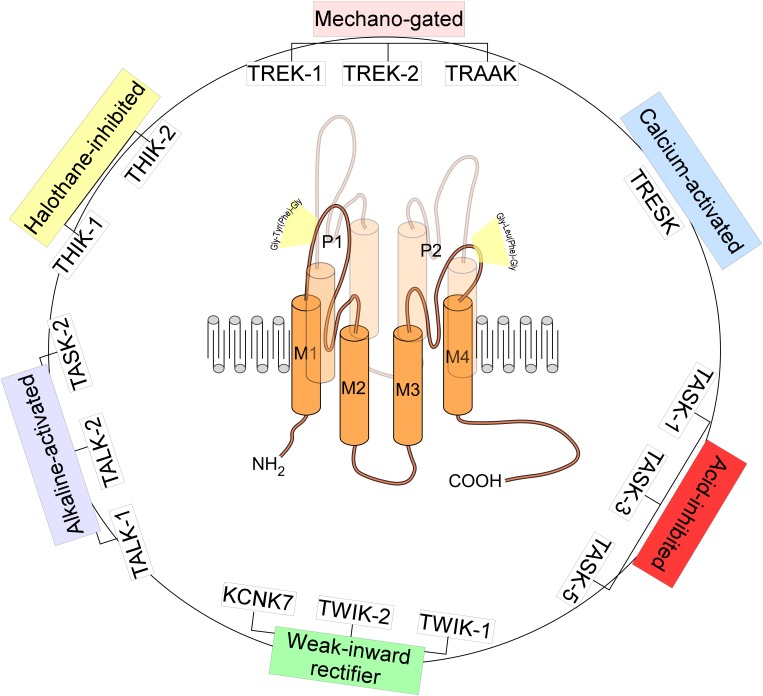
Structure and classification of K_2P_ channels. The family of K_2P_ channels is composed of 15 members grouped in six subfamilies. K_2P_ channels are two-pore domain potassium channels and the most recent class of K^+^ channels discovered. They assemble as dimers of four transmembrane segments (M1–M4) and two-pore domain (P1 and P2). They have an extended M1-P1 extracellular loop and cytosolic N- and C-termini. K_2P_ channels have a unique pore signature sequence Gly-Tyr(Phe)-Gly in the 1st pore (P1) and Gly-Leu(Phe)-Gly in the 2nd pore (P2).

**FIGURE 2:**
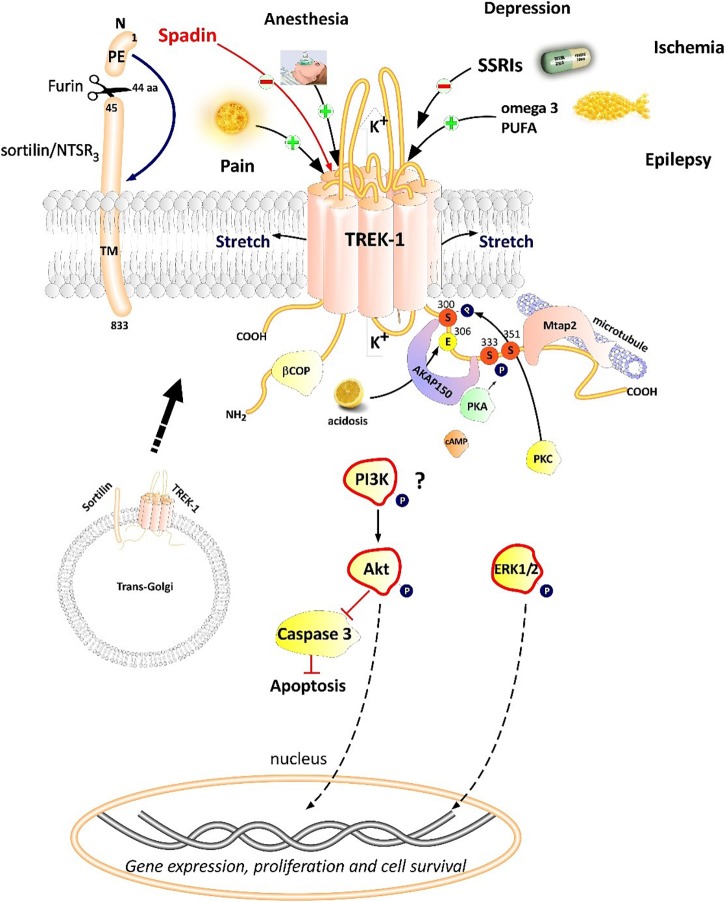
Polymodal TREK-1 regulation. TREK-1 is multiregulated by a variety of physical and chemical stimuli. TREK-1 possesses different protein partners such as AKAP150, β-COP, Mtap2, and sortilin. Sortilin interacts with TREK-1 an address it to the plasma membrane. Spadin is a synthetic peptide derived from sortilin which was shown to block TREK-1 with high affinity. Spadin antidepressant activity appears to be mediated through PI3K and Akt activation. TREK-1 is involved in numerous CNS pathologies such as depression, ischemia, epilepsy and pain.

**FIGURE 3:**
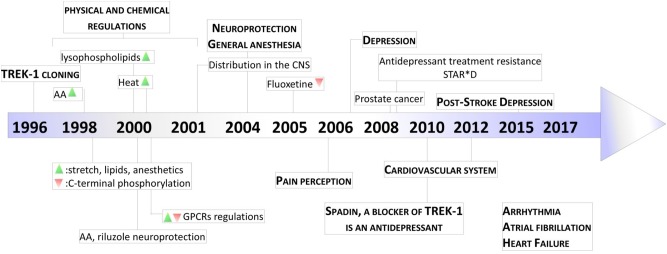
Key milestones in discovery of TREK-1 channels. The scheme presents the major dates from the cloning of TREK-1 channels to the discovery of its role in physiology and pathology. AA, arachidonic acid; GPCR, G-protein Coupled Receptor; STAR^∗^D, Sequenced Treatment Alternatives to Relieve Depression. Green and Red triangles represent TREK-1 activation and inhibition processes, respectively.

TREK-1, named KCNK2 or K_2P_2.1 belongs to a large family of K_2P_ channels containing 15 members grouped in six subfamilies. K_2P_ channels are the most recent class of K^+^ channels discovered. K_2P_ channels or the two-pore domain potassium channels are tandems of four transmembrane segments (M1–M4) containing two-pore domain (P1 and P2) (Figure 1, 2). They possess an extended M1-P1 extracellular loop and cytosolic N- and C-termini. K_2P_ channels have a unique pore signature sequence Gly-Tyr(Phe)-Gly in the 1st pore (P1) and Gly-Leu(Phe)-Gly in the 2nd pore (P2) (Honore, 2007; Figure 1). TREK-1 was first cloned from the mouse brain (Fink et al., 1996; Figure 3). TREK-1 was named after TWIK-1 channel the first cloned K_2P_ channel (Lesage et al., 1996). TREK-1 shares 28% sequence homology with TWIK-1 channel. TREK-1 is highly expressed in brain and lung, but is also present in kidney, heart and skeletal muscle. When we look at the brain localization, TREK-1 is highly expressed in several regions of the brain such as the olfactory bulb, the hippocampus, the cerebellum and the cortex (Fink et al., 1996).

TREK-1 displays an outward rectification in symmetrical K^+^ condition due to an external Mg^2+^ block present at negative potential and to a voltage-dependence mechanism (Maingret et al., 2002). Despite the absence of a voltage-sensing domain in K_2P_ channels, TREK-1 and some other K_2P_ channels show a strong voltage-dependency. The provenance of this voltage sensitivity comes from an ion-flux gating mechanism and the movement of three to four K^+^ ions into the high electric field of an inactive selectivity filter (Schewe et al., 2016). However, this voltage-dependency is switched off by physiological stimuli such as arachidonic acid (AA) and Phosphatidylinositol bisphosphate (PIP_2_), which convert TREK-1 to classical leak channels (Schewe et al., 2016). TREK-1 with TREK-2 and TRAAK are mechano- and thermo-sensitive K_2P_ channels (Honore, 2007). They are opened by stretch and cell swelling. TREK-1, TREK-2, and TRAAK knock-out mice are hypersensitive to mechanical force, they show mechanical allodynia and hyperalgesia during inflammation (Brohawn, 2015). It was reported that the mechanical force is transmitted to TREK-1 and TRAAK directly through the lipid bilayer (Brohawn et al., 2014). These channels open rapidly in response to tension and have a low threshold and broad range of tension activation (Brohawn, 2015).

### Physiological Regulation of TREK-1 Channels

TREK-1 channel is regulated by various physical and chemical stimuli (Figure 2). TREK-1 is opened by mechanical stretch in cell-attached and inside-out configurations in both COS transfected cells and oocytes (Patel et al., 1998). TREK-1 opening occurs at all positive and negative potentials. When the cytoskeleton is disrupted with colchicine or cytochalasin D, TREK-1 activation by stretch was not altered (Patel et al., 1998). TREK-1 was shown to be sensitive to temperature variation in different type of cells expressing TREK-1. Heat gradually and reversibly activates TREK-1 reaching a maximum of activation at 37°C. However, at 12°C TREK-1 basal current is suppressed (Maingret et al., 2000). TREK-1 heat activation needs cell integrity and cytosolic components since TREK-1 is insensitive to heat when patch membrane is excised (Maingret et al., 2000). TREK-1 is activated by polyunsaturated fatty acids (PUFA) such as AA in different patch-clamp configurations: whole-cell, cell-attached, inside-out and outside-out configurations (Patel et al., 1998). AA activates TREK-1 with a dose-dependent manner and requires C-terminal part of the channel which is crucial for the activation and the inhibition following phosphorylation by the protein kinase A (PKA). PIP_2_ stimulates native striatal TREK-1 current in inside-out patch configuration (Chemin et al., 2005). In Xenopus oocytes expressing TREK-1, PIP_2_ hydrolysis inhibits TREK-1 channel by modulating its voltage dependence (Lopes et al., 2005). It is also known that internal acidosis induces an activation of TREK-1 channels with the involvement of C-terminal part (Maingret et al., 1999). TREK subfamily members including TREK-1, TREK-2, and TRAAK are sensitive to phosphatidic acid (PA) which results from the hydrolysis of phosphatidylcholine by phospholipase D (PLD). It was shown that TREK-1 and TREK-2 but not TRAAK are positively regulated by PLD2 which selectively binds to their C-terminal domains (Comoglio et al., 2014).

### TREK-1 Partner Proteins

Proteomic approach based on immunoprecipitation and mass spectrometry analysis of native channel complexes allowed the identification of a scaffolding protein called AKAP150 (Sandoz et al., 2006; Figure 2). A-kinase anchoring protein (AKAP) anchors the regulatory subunit of PKA in proximity of substrates. AKAP150 forms signaling complexes within the neuron, these complexes are composed of PKA, PKC, PP2B (protein phosphatase 2B), PSD-95, SAP97 and ion channels (Esseltine and Scott, 2013). When anchored by AKAP150 within the post-M4 region, TREK-1 becomes an active leak channel insensitive to the stimuli, internal acidification AA, or mechanical stretch (Sandoz et al., 2006). The microtubule associated protein Mtap2 is another protein reported to associate with TREK-1 in the brain (Sandoz et al., 2008). Mtap2 binds to microtubules and stabilizes them. TREK-1 and Mtap2 colocalize in several brain regions such as the hippocampus, cerebellum, olfactory bulb, striatum and cortex. Similarly to AKAP150, Mtap2 binding to TREK-1 increases its activity (Sandoz et al., 2008). This increase in TREK-1 current amplitude is not due to a direct interaction with Mtap2 but results from an increase in the expression level of the channel at the plasma membrane. Within the neuron, TREK-1, AKAP150 and Mtap2 are simultaneously found in the post-synaptic terminals. This signaling complex regulates TREK-1 channel activity and its trafficking to the plasma membrane.

Using the yeast two-hybrid technique of the human cDNA library on the N-terminal region of TREK-1, a direct β-COP-TREK-1 interaction was reported (Kim et al., 2010). β-COP is a subunit of Coat Protein Complex I (COPI) whose role is to form coated vesicles and manage the retrograde traffic from the Golgi back to the endoplasmic reticulum (ER) or between different compartments inside the Golgi (Gomez-Navarro and Miller, 2016). β-COP depletion was found to decrease membrane expression of the cystic fibrosis transmembrane conductance regulator (CFTR) channel. Mutations in this chloride channel cause cystic fibrosis lung disease (Rennolds et al., 2008). β-COP increases TREK-1 surface expression and current amplitude. β-COP is involved in the forward transport of TREK-1 through a direct interaction with N-terminal part of the channel (Kim et al., 2010).

The most recently discovered protein partner of TREK-1 is sortilin (Petersen et al., 1997; Figure 2), also known as neurotensin receptor-3 (NTSR_3_) (Mazella et al., 1998). Sortilin is synthetized as a precursor called prosortilin which is cleaved in the trans-Golgi compartment by the proprotein convertase furin to generate a mature sortilin and the release of a 44 amino-acid named the propeptide (PE) (Munck Petersen et al., 1999). NTSR_3_ is made of a large luminal domain, a single transmembrane segment and a short C-terminal moiety. The amount of NTSR_3_ expressed at the plasma membrane does not exceed 10% while the majority is expressed intracellularly and involved mainly in intracellular trafficking. Both NTSR_3_ and TREK-1 are markedly expressed in the prefrontal (PFC) and cingulate cortex, the amygdala, the hippocampus, the nucleus accumbens, the dorsal raphe nucleus (DRN) and the hypothalamus. The expression of TREK-1 was enhanced at the plasma membrane in COS-7 cells co-transfected with NTSR_3_ (Mazella et al., 2010).

### TREK-1 Heterodimerization

The first K_2P_ member cloned TWIK-1 (Lesage et al., 1996), is expressed with TREK-1 in astrocytes (Zhou et al., 2009). The expression of a functional TWIK-1 channel necessitates its heterodimerization with TREK-1 to mediate the passive conductance in astrocytes (Hwang et al., 2014). As cited earlier in this review, TREK subfamily includes TREK-1, TREK-2 and TRAAK. Despite 78% of homology between TREK-1 and TREK-2, these two lipid- and mechano-activated K^+^ channels are differently regulated. Recent studies reported that functional TREK-1 heterodimers are formed with TREK-2 and TRAAK channels (Blin et al., 2016; Levitz et al., 2016). The heterodimer TREK-1-TREK-2 had minimal activity at physiological pH. However, TREK-1-TREK-2 complex is activated in both alkaline and acidic environments. TREK-1-TRAAK heterodimer shows activation by both intracellular acidification and alkalinization (Levitz et al., 2016). Functional TREK-1-TRAAK heterodimers are formed and present unique biophysical properties and different type of regulations (Blin et al., 2016). Heteromerization of TREK subfamily leads to diversification of K_2P_ channels and provides different kinds of regulations. Recently, it has been shown that a truncated form (TRESK-MT) of TRESK channel, a K_2P_ frequently associated with migraine, heteromerizes with and inhibits TREK-1 and TREK-2 (Royal et al., 2019). The heterodimer TRESK-TREK acts as a dominant negative increasing trigeminal sensory neuron excitability and migraine-like phenotype in mice (Royal et al., 2019).

### Pharmacology of TREK-1 Channels

#### TREK-1 Blockers

Since the discovery of the involvement of TREK-1 in several CNS pathologies, very high interest has been devoted to find molecules modulators of its activity. As depicted in Table 1, search for blockers of TREK-1 activity started with the discovery of the role of TREK-1 in depression processes. Mice model of depression lacking *kcnk2* (the gene encoding for TREK-1) displays a phenotype resistant to the development of depression (Heurteaux et al., 2006). Spadin was the first molecule that was developed in the aim to block TREK-1 channel and mimic the antidepressant-like phenotype of *kcnk2^-/-^* mice (Mazella et al., 2010; Djillani et al., 2018). Spadin is a peptide synthetized from the endogenous PE released after the cleavage of prosortilin by furin. Several analogs of spadin were developed afterward to improve spadin *in vivo* stability and antidepressant activity (Veyssiere et al., 2015; Djillani et al., 2017). From a library of 22 peptides, the shorter peptide sequence PE 22–28 exhibited higher inhibition potency for TREK-1 associated with an improved stability *in vivo* and antidepressant properties (Djillani et al., 2017). Similar properties were found in its biotinylated product or analog G/A-PE 22–28 (where Gly is replaced by an Ala). These spadin analogs show an extremely high affinity for TREK-1 as inhibition occurs at nanomolar concentrations. More interesting, spadin and analogs are specific blockers for TREK-1 as no effect was observed on both TREK-2 and TRAAK, the two closest K_2P_ members to TREK-1 which belong to the same subfamily and share almost 80% of homology (Moha Ou Maati et al., 2012; Djillani et al., 2017).

**Table 1:** TREK-1 blockers.

	Molecule	IC50	Cell type	Specificity	References
Peptides	Spadin (PE 12–28)	70 nM	COS, HEK, hTREK-1/HEK	Specific for TREK-1 channels, no effect on TREK-2, TRAAK, TASK or TRESK channels	Mazella et al., 2010
	PE 22–28	0.12 nM	hTREK-1/HEK		Djillani et al., 2017
	Biotin-PE 22–28	nd	hTREK-1/HEK		Djillani et al., 2017
	G/A-PE 22–28	0.1 nM	hTREK-1/HEK		Djillani et al., 2017
	Biotin-G/A-PE 22–28	1.2 nM	hTREK-1/HEK		Djillani et al., 2017
SSRIs	Fluoxetine	19 μM	tsA 201 cells	Antagonize TREK-2 (IC50 = 28.7 ± 7.6 μM), SERT (Ki = 0.07 μM), NAT (Ki = 8–10 μM) and Nav1.5 (IC50 = 39.4 ± 2 μM)	Kennard et al., 2005; Wong et al., 2005; Poulin et al., 2014
	Norfluoxetine	9 μM	tsA 201 cells	Blocks TREK-2 (IC50 = 4.9 ± 0.5 μM), Nav1.5 (IC50 = 29.5 ± 1 μM)	Kennard et al., 2005; Poulin et al., 2014; Mcclenaghan et al., 2016
	Paroxetine	20 μM tested	HEK293 cells	blocks TREK-2 (at 20 μM tested), SERT (Kd = 0.13 nM), GIRK and Kv3.1	Hirano et al., 2005; Kim et al., 2017
	Citalopram	100 μM tested	HEK293 cells	blocks TREK-2 (at 100 μM tested), SERT, Kv1.5 (IC50 = 2.8 ± 1.1 μM) and L-type Ca^2+^ channels (IC50 = 60.3 ± 8.5 μM)	Kim et al., 2017
	Escitalopram	81.94 μM	HEK293 cells	nd	Lin et al., 2015
Antipsychotics	Fluphenazine	4.7 μM	COS cells	Block TREK-2 (at 10 μM tested), no effect on TRAAK channels at 10 μM	Thummler et al., 2007
	Chlorpromazine	2.7 μM			
	Haloperidol	5.5 μM			
	Flupenthixol	2 μM			
	Loxapine	19.7 μM			
	Pimozide	1.8 μM			
	Clozapine	10 μM tested			
DHP Ca^2+^ channel antagonists	Amlodipine	0.43 μM	Bovine AZF	Block L-type Ca^2+^-channels	Liu et al., 2007
	Niguldipine	0.75 μM			
Other TREK-1 blockers	SID1900	29.72 μM	HEK293 cells	nd	Ye et al., 2015
	l-NBP, d-NBP, ld-NBP	0.06 ± 0.03 μM	CHO cells	nd	Ji et al., 2011
	L-methionine	1 mM	Bladder smooth muscle	nd	Lei et al., 2014
	Lig4-4	2.06 μM	CHO cells	Blocks Kv2.1, Kv1.5, Kv3.1, hERG and neuronal Na^+^ and Ca^2+^channels (IC50 = 30 μM)	Wang et al., 2018


Selective Serotonin Reuptake Inhibitors (SSRI) is a family of small molecules with a high affinity for the serotonin transporter, SERT (Hirano et al., 2005). SERT blockade generates an accumulation of serotonin in the synaptic clefts and displays antidepressant effects in clinic. In addition, at clinical concentrations, SSRI and fluoxetine antagonize TREK-1 channel (IC_50_ = 19 μM for fluoxetine and IC_50_ = 9 μM for Norfluoxetine, its active metabolite) (Kennard et al., 2005). Other SSRIs such as paroxetine, citalopram or escitalopram also block TREK-1 with different potencies. However, SSRIs are not specific for TREK-1 since they target TREK-2, Nav1.5, and L-type Ca^2+^ channels (Wong et al., 2005; Poulin et al., 2014; Mcclenaghan et al., 2016).

Antipsychotic drugs used to treat psychosis, like schizophrenia and bipolar disorder, were shown to be potent blockers of TREK-1 channels. They antagonize TREK-1 and TREK-2 but do not affect TRAAK channels (Table 1; Thummler et al., 2007). TREK-1 blockade was observed with both typical and atypical antipsychotic. For example, chlorpromazine and Loxapine block dose-dependently TREK-1 channels with IC_50_ of 2.7 and 19.7 μM (Thummler et al., 2007). These data demonstrate a potential link between TREK-1, schizophrenia and bipolar disorder.

Dihydropyridine analogs such as amlodipine or niguldipine are L-type Ca^2+^ channel blockers. It has been shown that they also block TREK-1 with high affinity (IC_50_ = 0.43 and 0.75 μM for amlodipine and niguldipine, respectively). In addition, mice lacking the L-type channel Cav1.3 display an antidepressant-like phenotype (Busquet et al., 2010). The compound SID1900 blocks TREK-1 with an IC_50_∼30 μM and was shown to produce antidepressant-like properties in a rat model of chronic unpredictable mild stress (CUMS) (Ye et al., 2015). SID1900 effect was comparable to spadin. Still there is no evidence about other possible targets of SID1900 and more data are needed to conclude about its specificity. Finally, other molecules were reported to block TREK-1 channels and provide neuroprotection against ischemia and stroke such as 3-n-butylphtalide (NBP) and its analog lig4-4 (Ji et al., 2011; Wang et al., 2018). However, if information is lacking about the specificity of NBP, lig4-4 was reported to affect hERG channel, voltage-gated K^+^ channels (K_v_), neuronal Na^+^ and Ca^2+^ channels, which could limit its development as potential neuroprotective molecule.

#### TREK-1 Activators

TREK-1 channel is activated by volatile general anesthetics such as chloroform, diethyl ether, halothane and isoflurane and it was shown that the C-terminal part is critical for its activation (Table 2; Patel et al., 1999). If the chloroform seems to be selective for TREK-1 channels with an EC_50_ (0.2–1.6 mM), halothane and isoflurane activate both TREK-1 and TASK channels. Furthermore, diethyl ether opens TREK-1 but decrease TASK channel activity (Patel et al., 1999). In contrast to general anesthetics, local anesthetics like bupivacaine behave as inhibitors of TREK-1 (Kindler et al., 1999). AA and other PUFAs open TREK-1 channels in dose-dependent manner (Patel et al., 1998). Channel opening by AA, α-linolenic acid (ALA), and docosahexaenoic acid (DHA) is thought to contribute to neuroprotection (Lauritzen et al., 2000). If SSRI antidepressants and antipsychotics drugs act as TREK-1 blockers, mood stabilizers such as lithium chloride and antiepileptics drugs like gabapentine, valproate and carbamazepine were reported to activate TREK-1 channel (Kim et al., 2017). The tetrazole based compound BL-1249 showed an interesting TREK-1 activation with an EC_50_ of around 1.5 μM *in vitro* in cultured human urinary bladder myocytes (Tertyshnikova et al., 2005). In a pancreatic carcinoma cell line, BL-1249 activates dose-dependently TREK-1 with an IC_50_ = 2 ± 2 μM (Sauter et al., 2016). Recent studies provided more insights into the action mechanism of BL-1249. Indeed, it was shown that BL-1249 activates the selectivity filter C-type gate and activates selectively TREK-1 and TREK-2 channels 10-fold stronger than TRAAK channels (Pope et al., 2018). In addition, BL-1249 action requires the C-terminal tail of the channel for the activation and the transmembrane domains M2 and M3 are critical for its selectivity (Pope et al., 2018). Following the screening of more than 106,000 small molecules, an activator of TREK-1 named ML67 and its optimized analog ML67-33 were identified (Bagriantsev et al., 2013). Then after, two molecules with higher potency were recently developed by the same laboratory, ML335 and ML402 which potentiate TREK-1 activity with EC_50_ of 5.2 ± 0.8 μM and 5.9 ± 1.6 μM, respectively (Table 2; Lolicato et al., 2017). ML335 and ML402 bind and activate a cryptic binding pocket within the C-type gate selectivity filter of TREK-1 channel (Lolicato et al., 2017). Non-steroid anti-inflammatory drugs (NSAIDs) such as flufenamic acid, niflumic acid and mefenamic acid were reported to activate TREK-1 channels independently of cyclooxygenase COX inhibition (Veale et al., 2014). Caffeic acid derivatives were reported to activates TREK-1 channel and provide potent analgesic effect *in vivo* (Rodrigues et al., 2014; Vivier et al., 2017). Finally, small molecule GI-530159 was described as TREK-1 opener which reduces small DRG neurons excitability (Loucif et al., 2018). However, GI-530159 is not selective for TREK-1 channels since it activates also TREK-2 with no significant effect on TRAAK channels (Loucif et al., 2018).

**Table 2:** TREK-1 activators.

	Molecule	EC50	Cell type	Specificity	References
Neuroprotective agents	Riluzole	Transient activation (100 μM tested)	COS cells	Activates TRAAK (EC50∼60 μM), blocks TTX-sensitive (IC50 = 90 μM) and TTX-resistant (IC50 = 143 μM) sodium channels. It antagonizes Ca^2+^ entry induced by glutamic acid (IC50 = 0.30 ± 0.07 μM) or by NMDA (IC50 = 12.8 ± 2.4 μM). Riluzole inhibits glutamate release in the caudate nucleus (10 μM tested) and the hippocampus (10–30 μM tested)	Cheramy et al., 1992; Martin et al., 1993; Hubert et al., 1994; Song et al., 1997; Duprat et al., 2000
Mood stabilizers	Lithium	Maximum of activation at 1 mM	HEK293 cells	nd	Kim et al., 2017
	Gabapentin	100μM tested		nd	Kim et al., 2017
	Valproate	100 μM tested		nd	Kim et al., 2017
	Carbamazepine	Maximum of activation at 100 μM		nd	Kim et al., 2017
Carbazole-based	ML67	EC50 = 213 ± 1.2 μM	Xenopus oocytes	Activates TREK-2 (EC50∼250 μM) and TRAAK	Bagriantsev et al., 2013
	ML67-33	EC50 = 36.3 ± 1 μM	Xenopus oocytes	Activates TREK-2 (EC50 = 30.2 ± 1.4 μM) and TRAAK (EC50 = 27.3 ± 1.2 μM)	
		EC50 = 9.7 ± 1.2 μM	HEK293		
	ML335	EC50 = 14.3 ± 2.7 μM	Xenopus oocytes	Activates TREK-2 (EC50 = 5.2 ± 0.5 μM), no effect on TRAAK	Lolicato et al., 2017
		EC50 = 5.2 ± 0.8 μM	HEK293		
	ML402	EC50 = 13.7 ± 7 μM	Xenopus oocytes	Activates TREK-2 (EC50 = 5.9 ± 1.6 μM), no effect on TRAAK	
		EC50 = 5.9 ± 1.6 μM	HEK293		
Polyunsaturated fatty acids (PUFA)	Arachidonic acid	(0.1–10 μM) tested	COS cells	Activates TREK-2 (1–10 μM), no effect on TRAAK (0.01–100 μM)	Patel et al., 1998
General volatile anesthetics	Chloroform	(0.2–1.6 mM)	COS cells	Selective for TREK-1	Patel et al., 1999
	Halothane	(0.04–4 mM)		Activates TASK channels	
	Isoflurane	2 mM tested		Activates TASK channels	
	Diethyl ether	0.8 mM tested		Decrease TASK channel activity	
Analgesics (NSAIDs)	Fenamate	EC50∼100 μM	COS cells	Activates TREK-2 (EC50∼100 μM) and TRAAK (>100 μM)	Takahira et al., 2005
	Flufenamic acid	250 ± 35% at 100 μM	TsA201 cells	nd	Veale et al., 2014
Tetrazole-based	BL-1249	EC50 = 1.49 ± 0.08 μM	Cultured human urinary bladder myocytes	nd	Tertyshnikova et al., 2005
		EC50 = 2 ± 2 μM	Pancreatic ductal adenocarcinoma	nd	Sauter et al., 2016


#### TREK-1 Modulators

Riluzole (Table 2) is a neuroprotective molecule marketed as an anticonvulsant drug. Its action mechanism involves the blockade of glutamate receptors. It is also prescribed to prolong the survival of patient suffering from amyotrophic lateral sclerosis. Besides, riluzole was shown to act as a transient TREK-1 activator within 30 s and strong inhibitor after 90 s (Duprat et al., 2000). The dual activity on TREK-1 is thought to be mediated through cyclic Adenosine MonoPhosphate (cAMP) activation of PKA. On the other hand, riluzole produces a sustained activation of TRAAK without an inhibition.

### TREK-1 and CNS Disorders

TREK-1 channels show a widespread distribution in rat and mouse brains (Hervieu et al., 2001; Talley et al., 2001). It is rational to imagine multiple roles that TREK-1 can play in the CNS. In this part of the review, we will explain in depth why TREK-1 channel became a very promising target in numbers of pathologies that affect the CNS (Figure 3).

#### TREK-1 in Depression

The role of TREK-1 in depression was demonstrated in mice invalidated for *kcnk2*, the gene encoding for TREK-1 (Heurteaux et al., 2006). TREK-1 knock-out mice displayed a phenotype resistant to depression in five different mouse models of depression: the Porsolt forced swim test (FST), the tail suspension test (TST), the conditioned suppression of motility test (CSMT), the learned helplessness test (LH), and the novelty-suppressed feeding test (NSF) (Table 3; Heurteaux et al., 2006). The behavior of *kcnk2^-/-^* mice was similar to those treated acutely or chronically with classical antidepressants like fluoxetine (Heurteaux et al., 2006). *Kcnk2*-deficient mice show an increase in serotonin (5-HT) neurotransmission in the DRN neurons. Deletion of TREK-1 increases neurogenesis induced with a chronic treatment with antidepressants (Heurteaux et al., 2006). Blocking TREK-1 has become a novel strategy to design new generation of antidepressants. At the concentrations used clinically, SSRIs inhibits TREK-1 channels (Kennard et al., 2005).

**Table 3:** TREK-1 in CNS pathologies.

Pathology	TREK-1 expression			Potential treatment	References
			
	Immunofluorescence	mRNA	Protein		
Depression	nd	nd	↑ in the frontal cortex 3 weeks after CMS in rat No change in the hippocampus	TREK-1 blockers as potential treatment in depression	Chen et al., 2015
			Fluoxetine per os daily for 3 weeks		
			Reverse TREK-1 overexpression in the frontal cortex		
Ischemia	Broad distribution in the cortex and CA1 (glia and neurons)	↑ in the hippocampus at 3 and 30 days after BCAL *In situ* hybridization: ↑ in the cortex and hippocampus 7 and 30 days after BCAL (Xu et al., 2004)	in the hippocampus 7 and 30 days after MCAO	TREK-1 openers as potential treatment in ischemia	Wang et al., 2012
	↑ in the cortex and the hippocampus after 3, 7, and 30 days after MCAO				
Pain perception	TREK-1 expression in DRG sensory neurons	↑ in DRG of CCI mice	↑ in DRG of CCI mice	TREK-1 openers as potential analgesics	Alloui et al., 2006; Han et al., 2016
Post-stroke depression	Broad distribution in PFC, CA1, CA3, and DG	**No treatment, after 31 days of MMCAO**			Lin et al., 2015
		↑ in PFC, CA3 and DG	↑ in PFC, CA1, CA3 and DG	TREK-1 blockers as potential treatment in PSD	
		**Escitalopram (3 weeks)**			
		↓ in PFC, CA3 and DG	↓ in PFC, CA1,CA3 and DG		


Spadin was previously described as a specific blocker of TREK-1 with a high affinity (IC_50_ = 40–70 nM) (Mazella et al., 2010; Djillani et al., 2017, 2018). Spadin consists in 17-amino acid peptide synthesized from the propeptide (PE). PE is 44 amino acids generated from the maturation of sortilin by the cleavage of the prosortilin with furin (Munck Petersen et al., 1999). Spadin binds with high affinities both TREK-1 and sortilin/neurotensin receptor 3 (NTSR3). Spadin by blocking TREK-1 generates mice with depression-resistance phenotype in several depression tests after only 4 days of treatment. Similarly to *kcnk2*^-/-^ mice, spadin enhances 5-HT neurotransmission in DRN serotonergic neurons (Mazella et al., 2010). In *in vitro* experiments, spadin stimulates MAPK and PI3K pathways in time and concentration-dependent manner. Spadin at 100 nM increases the phosphorylation of ERK1/2 and Akt but does not affect the phosphorylation of mTOR, suggesting an original mechanism of action of spadin different from the other fast-acting antidepressant ketamine which was demonstrated to be mTOR-dependent (Devader et al., 2015). Spadin has protective effects on neurons against staurosporine-induced Caspase-3 apoptosis through the specific activation of the PI3K pathway. Spadin increases transiently and after 8 h the mRNA expression of PSD-95 and synapsin, two markers of synaptogenesis in the brain. This increase is associated with a transient BDNF increase only after 5 h. Spadin increases also the proportion of mature spines in cortical neuronal culture. *In vivo*, daily spadin administration for 4 days increased mRNA expression of BDNF, PSD-95 and synapsin after only 7 days in the hippocampus. However, in the prefrontal cortex (PFC), only BDNF was enhanced after 3 weeks (Devader et al., 2015).

The FST antidepressant activities of spadin disappear after 7 h after an acute treatment. To improve the bioavailability and the *in vivo* stability of spadin, retro-inverso analogs of spadin were designed and screened on the cell line stably expressing the human TREK-1 (Veyssiere et al., 2015). Two analogs, 3 and 8, were identified. They prolong the antidepressant activity from 7 to 16 h in FST. However, due to some issues concerning *in vitro* toxicity of analog 3 and 8 at higher concentrations, other strategies were conducted in order to ameliorate the benefit/risk ratio. By shortening spadin sequence, a peptide PE 22-28 containing only 7 amino acids was identified as the shortest efficient molecule displaying an antidepressant activity (Djillani et al., 2017, 2018). The role of TREK-1 in mice was supported by other studies in human like STAR(^∗^)D which highlights a strong association between resistance to SSRIs and the existence of four single nucleotide polymorphisms (SNP) in *kcnk2* gene (Perlis et al., 2008). Another SNP, rs6686529 located in *kcnk2* gene was found to be associated with major depression disorder and response to antidepressant treatment (Liou et al., 2009). All these data highlight the key role of TREK-1 in depression and the need to design blockers of TREK-1 as promising original antidepressant (Figure 3).

#### TREK-1 in Neuroprotection

##### TREK-1 in epileptogenesis

TREK-1 is expressed in GABA-containing interneurons in the cortex and the hippocampus (Hervieu et al., 2001). *In vivo*, It was reported that TREK-1 activators PUFA such as α-linolenic acid protect rats treated with the glutamate receptor agonist, kainic acid (KA) against seizures and hippocampal lesions (Lauritzen et al., 2000). Moreover, PUFA neuroprotection was observed in another model of seizures using glutamatergic neurons. This effect was associated with the inhibition of the glutamatergic neurotransmission (Lauritzen et al., 2000). TREK-1 deficient mice were shown to be more vulnerable to develop epileptic seizures triggered by KA and pentylenetetrazol (PTZ, GABA_A_ receptor antagonist) (Heurteaux et al., 2004). The expression of *c-fos*, a marker of neuronal excitability was increased in CA3 pyramidal neurons following treatment with KA (Heurteaux et al., 2004). Linolenic acid and lysophosphatidylcholine (LPC) decrease KA-induced seizures in wild-type mice. However, no effect was observed in mice lacking TREK-1 channels (Heurteaux et al., 2004). The protective effects of linolenic acid and LPC require the presence of TREK-1 channels. Status epilepticus (SE) is a persistent repeated seizure activity for more than 5 min. Development of TREK-1 mutant (TREK-M) produced a constitutively open TREK-1 channels that showed resistance to PKA and PKC downregulation. TREK-M was able to hyperpolarize the plasma membrane and decrease spontaneous firing of hippocampal neurons in culture (Dey et al., 2014). *In vivo* administration of a recombinant adenoassociated virus (AAV)-mediated delivery of TREK-M in both entorhinal cortex and hippocampal CA3 regions, reduced by 50% the duration of SE in a mouse model of SE induced with lithium and pilocarpine. AAV-TREK-M prevented neuronal death in both entorhinal cortex and CA3 after injection (Table 3; Dey et al., 2014). Since TREK-1 opening results in neuroprotection against epileptic episodes, blocking this AA-activated K_2P_ channel could in contrast be deleterious. Surprisingly, TREK-1 antagonist spadin does not enhance seizures induced by KA or PTZ when injected to mice. More interestingly, mice treated with spadin show more resistance to develop generalized convulsions and to induce death (Moha Ou Maati et al., 2012; Djillani et al., 2018).

##### TREK-1 in ischemia

Using a model of global ischemia, results from transient bilateral occlusion of common carotid arteries in wild-type and knock-out TREK-1 mice showed that 74% of TREK^-/-^ mice died 3 days after ischemia whereas only 34% died in wild-type group (Heurteaux et al., 2004). Here again, linolenic acid or LPC pretreatment were unable to protect against ischemia in TREK-1 deficient mice in contrast to wild-type mice in which mice survival was significantly increased (Heurteaux et al., 2004). Spinal cord ischemia is induced in mice by occluding both the aortic arch and left sub-clavian artery. It was reported that 75% of *kcnk2*^-/-^ mice died 3 h after 10 min ischemia compared to only 14% of wild-type mice 24 h after ischemia (Heurteaux et al., 2004). Furthermore, surviving TREK-1^-/-^ mice developed severe hind limb paralysis. However, no neurological deficit was observed with wild-type mice (Heurteaux et al., 2004). In addition to neurons, astrocytes were shown to play an important role in brain ischemia. TREK-1 contributes in maintaining the highly negative membrane potential in astrocytes which is crucial in controlling cell excitability (Zhou et al., 2009). In cultured cortical astrocytes and in hippocampal slices, TREK-1 is widely expressed. Cortical and hippocampal TREK-1 channels were upregulated during astrogliosis following focal ischemia (Wang et al., 2012). In hypoxic conditions, TREK-1 protein expression in astrocytes was also upregulated which increases glutamate clearance, suppressed the astrocytic marker S100β and block neuronal death (Table 3; Wu et al., 2013). Glutamate is the main excitatory neurotransmitter in the brain. Astrocytes release glutamate following GPCR activation. DH Woo et al. demonstrate that TREK-1 channel is involved in the fast glutamate release which requires the interaction between the N-terminal domain of TREK-1 and the G_βγ_ of the G_αi_PCR (Woo et al., 2012).

##### TREK-1 in general anesthesia

In general anesthesia, at clinical doses used for isoflurane, diethyl ether, halothane and chloroform, TREK-1 channels are activated (Patel et al., 1999). TREK-1 channel activation implies the C-terminal moiety and leads to cell hyperpolarization, decreased action potential firing and neuroprotection. The laughing gas nitrous oxide and xenon mostly exert their anesthetic effects via antagonizing NMDA receptors since they provide analgesia, euphoria and neuroprotection. They do not potentiate GABA_A_ receptors (Gruss et al., 2004). Nitrous oxide and xenon were shown to open TREK-1 channels with Glu306 as a critical amino acid for the anesthetic activation of TREK-1 (Gruss et al., 2004). Chloral hydrate is used in pediatrics in certain forms of epilepsy such as progressive myoclonus and refractory childhood epilepsies. Upon administration, Chloral hydrate is rapidly metabolized into its active metabolite 2,2,2-trichlorethanol (TCE) which is thought to be responsible for the chloral hydrate activity (Harinath and Sikdar, 2004). TCE induces depression of the CNS by potentiating GABA_A_ receptors and inhibiting NMDA, AMPA, and Kainate receptors. It was reported that TCE also activates TREK-1 and TRAAK channels and contributes to the central anesthetic effect (Harinath and Sikdar, 2004). *In vivo*, mice lacking TREK-1 channels displayed a lower sensitivity to the most potent TREK-1 activators, chloroform and halothane and also to sevoflurane, desflurane and isoflurane (Heurteaux et al., 2004). The threshold to provoke anesthesia and the doses required for the anesthetic action were higher. Phenobarbital, a general anesthetic that does not modulate TREK-1 channels, fails to reproduce these effects in *kcnk2*^-/-^ mice. Thus, the observed effects were due specifically to TREK channel absence. Recently, *in vitro* and *in vivo* preconditioning with sevoflurane was shown to provide neuroprotection through activation of TREK-1 channels (Tong et al., 2014). Rats treated with the anesthetic sevoflurane after MCAO display neuroprotection which seems to be mediated by TREK-1 channels (Table 3; Pan et al., 2017).

#### Role of TREK-1 in Post-stroke Depression

Given the neuroprotective role of TREK-1 channels in stroke and its involvement in the depression process. It makes sense to address the role of this K_2P_ channel in the physiopathology of post-stroke depression (PSD). Recently, several laboratories have started to investigate the potential role of TREK-1 channels in this pathology. PSD is a high prevalence neuropsychiatric disorder that takes place after stroke onset. It has been shown that TREK-1 channel expression levels were elevated in the hippocampus and the PFC in rat model of PSD (Table 3; Lin et al., 2015). Noteworthy, TREK-1 channel up-regulation was suppressed following chronic treatment with the TREK-1 blocker and the SSRI, escitalopram. In this PSD rat model, the main actors causing PSD in human patients were reproduced. For example, the middle cerebral artery occlusion (MCAO) surgery was set up to mimic stroke and chronic mild stress (CMS) and isolation rearing to mimic stress and psychosocial conditions, respectively (Lin et al., 2015).

#### TREK-1 in Pain Perception

TREK-1 channels are known to be modulated by heat. Those K_2P_ channels are gradually and reversibly opened by temperature. Noteworthy, TREK-1 channel opening is reversed by cAMP and prostaglandin E2, two sensitizers of peripheral and central thermoreceptors. TREK-1 blockade is due to the phosphorylation of Ser333 on its C-terminal end. The thermosensitive TREK-1 channel is highly expressed and localized in small dorsal root ganglion (DRG) neurons and in central hypothalamic neurons, areas that are highly involved in pain (Maingret et al., 2000). Sixty percent of sensory neurons express TREK-1 and most of them are associated with substance P. More than 40% of DRG neurons that express TREK-1 channels also express the thermal nociceptor TRPV1 channels. *In vivo*, compared to wild type mice, mice lacking *kcnk2* gene are highly sensitive to low-threshold thermal pain and mechanical stimuli (Alloui et al., 2006). TREK-1 deficient mice showed an exacerbated focal inflammatory response after spinal cord injury (SCI). Furthermore, TREK-1 deficiency enhanced astrogliosis, neuronal apoptosis, demyelination and retarded motor recovery (Fang et al., 2017). Nevertheless, in other reported studies, in the DRG, the microRNA miR-183 expression was decreased and TREK-1 expression was increased in neuropathic pain induced by chronic constriction of sciatic nerve (CCI) (Table 3; Han et al., 2016; Shi et al., 2018). Intrathecal injection of miR-183 alleviated pain in rat model of CCI and downregulated TREK-1 expression (Shi et al., 2018). Recently, it was reported that TREK-1 modulator riluzole prevents both sensory and motor deficits in neuropathic pain induced by the chemotherapy drug oxaliplatin (Poupon et al., 2018). Nevertheless, conclusions drawn by this paper are not totally convincing because authors only consider riluzole as an activator and do not take into account its inhibitory effects. Finally, TREK-2 and TRAAK channels, the other members of TREK subfamily are characterized in the small sized DRG neurons of rats (Viatchenko-Karpinski et al., 2018).

### Peripheral Roles of TREK-1 Channels

#### TREK-1 in the Heart

In the heart, the stretch-activated K^+^ channels (SAK) repolarize cell membrane and counterbalance the activity of stretch-activated Cation channels (SAC) which in contrast increase cation influx and cell depolarization. TREK-1 channel presents a serious candidate to form SAK in heart with the large conductance Ca^2+^-activated K^+^ channel (BK_Ca_) and K_ATP_ channels (Decher et al., 2017a). In rat heart, all K_2P_ channel genes were detected in at least one heart chamber with a prevalence of TWIK-2, TASK-1 and TREK-1 expression. TREK-1 channel was highly expressed in the right ventricle (Liu and Saint, 2004). In rat neonatal atrial myocytes, using single channel inside-out patch, TREK-1-like current was activated by AA and internal acidosis (Kim and Clapham, 1989). This TREK-1-like current was shown to be reversibly activated by volatile anesthetics such as chloroform, halothane and isoflurane. It was also downregulated by cAMP analogs and β-adrenergic receptor agonists (Terrenoire et al., 2001). SAK current shared identical biophysical properties with the recombinant TREK-1 current such as the single channel conductance, no voltage-dependency and sensitivity to volatile anesthetics. In the ventricular cardiomyocytes, two variants of TREK-1 were found, large conductance and small conductance with (132 ± 5 pS) and (41 ± 5 pS), respectively, at positive potentials (Xian Tao et al., 2006). The low-conductance TREK-1 variant was opened by mechanical stretch, internal acidification and AA. The biophysical properties of the two TREK-1 channels were similar to those displayed by the recombinant TREK-1 channels expressed in HEK293 cells. Using cardiac specific TREK-1 deficient mice, it was proposed that TREK-1 plays an essential role in sinoatrial node cell excitability (Unudurthi et al., 2016). Recently, a heterozygous point mutation in the selectivity filter of TREK-1 channel was identified in patients diagnosed with right ventricular outflow tract (RVOT) tachycardia (Decher et al., 2017b). More interestingly, TREK-M showed a hypersensitivity to stretch and permeability to sodium ions (Decher et al., 2017b; Figure 3).

#### Role of TREK-1 in Other Tissues

In addition to the central roles of TREK-1 channels in the brain, it was thoroughly rational to think that modulating TREK-1 channels could play a peripheral role as this K_2P_ channel is expressed in number of tissues such as pancreas, prostate and smooth muscle cells (Medhurst et al., 2001). The pancreas and particularly the insulin secreting β-cells constitute one of the main targets of the peripheral tissues where TREK-1 and other K_2P_ channels play an important role in glucose homeostasis (Kang et al., 2004; Mazella et al., 2010; Dadi et al., 2014; Vierra et al., 2015). Given the importance of TREK-1 channel as a key modulator of plasma membrane potential in addition to its regulation by various physical and chemical stimuli, its role in the glucose homeostasis has been investigated using spadin (Hivelin et al., 2016). Spadin inhibits TREK-1 channels in the pancreatic β-cell line β-TC3 (Mazella et al., 2010). It is known that the regulation of insulin secretion by pancreatic β-cell is fine-tuned by a variety of hormones such as glucagon like-peptide-1 (GLP-1), leptin, estrogen, melatonin and growth hormone (Fu et al., 2013). It is well established that ATP-sensitive potassium channels (K_ATP_) play a critical role in maintaining glucose homeostasis (Mctaggart et al., 2010). K_ATP_ channels serve as a metabolic sensor for the pancreatic β-cell. When glucose levels increase, K_ATP_ channels close, inducing a membrane depolarization resulting in the opening of Ca^2+^ channels as well as the secretion of numerous hormones and amplification of the effects of glucose and other secretagogues such as GLP-1 (Mctaggart et al., 2010). TREK-1 blockade by spadin induces insulin secretion only upon glucose stimulation. However, in contrast to incretin hormones, spadin induces Ca^2+^ increase and insulin release through an original PKA-independent mechanism (Hivelin et al., 2016). TREK-1 channels are inhibited as well by the PKA phosphorylation of the Ser333 at the C-terminal moiety of the channel (Murbartián et al., 2005), which is the consequence of the activation of different G-protein-coupled receptors at the plasma membrane. By binding to their GLP-1 receptors, GLP-1 and its analogs such as exendin-4 are able to trigger cAMP synthesis and thus induce PKA activation leading to insulin secretion (Göke et al., 1993). Then, insulin release by β-cells is potentiated by TREK-1 closing following PKA phosphorylation. Spadin or its analogs could substitute to incretin hormone actions and could propose a therapeutic alternative in case of inefficacy of antidiabetic treatment using GLP-1 mimetics. Among the other K_2P_ channels, the mechano-sensitive K_2P_ channel TREK-1 is predominately expressed in the human detrusor channel (Andersson and Arner, 2004). Recently, a study assessing the relationship between six SNP and the occurrence of overactive lower urinary tract symptoms (LUTS), revealed a strong association between TREK-1 polymorphisms (rs758937019-CT genotype and rs758937019-T allele) and overactive LUTS in human (Nedumaran et al., 2019).

## Conclusion and Perspectives

Since their cloning 20 years ago, the physiological importance of TREK-1 channels has continued to grow (Figure 3). Today, TREK-1 channels have been shown to be important and their presence is essential in a number of physiopathological processes. Their involvement in these different processes demonstrate the necessity to design pharmacological modulators, activators or inhibitors, of these channels to correct any TREK-1-related dysfunctions.

Despites a number of studies and many molecule screenings, only few putative new drugs were identified. The activators belonging to the ML and BL series show interesting results. However, they display lower affinities (in the micromolar range) and they are not specific for TREK-1 channels as they open other K_2P_ channels. TREK-1 openers are needed in pathologies such as ischemia, epilepsy and pain. The challenge is to improve their affinity, specificity and ability to cross the blood-brain barrier. The new ML and BL series of TREK- activators have not yet been tested in pathologies such epilepsy and ischemia. It would be interesting to investigate any neuroprotection effects of these molecules in rodent models of epilepsy or ischemia. In the same time, their specificity should be improved to avoid any off-target effects resulting from modulating other K_2P_ channels.

As inhibitors, the most promising molecules are spadin and its analogs. These peptides are very specific in the K_2P_ family and display very high affinities, in the picomolar range for the shorter analogs (Djillani et al., 2017). Depression is a complex mood disorder where different actors are involved. TREK-1 is definitely one of the most promising ion channel that plays a key role in the neurobiology of depression. TREK-1 blockers such as spadin and short analogs display a huge therapeutic value over the classical antidepressants prescribed nowadays. They are fast-acting with highly affinity and specificity for TREK-1. More interestingly, they cross the blood-brain barrier which facilitates their further optimization and development as antidepressant drugs useful in clinic.

In the future, in addition to looking for new TREK-1 modulators to treat neuropsychiatric disorders, it is relevant to study the role of TREK-1 channels in peripheral tissues and understand their involvement in physiopathology. An emerging area where TREK-1 is displaying a growing interest is the heart. TREK-1 expression has been shown to change in cardiac hypertrophy (Wang et al., 2013). Furthermore, a TREK-M was found to change channel permeability to sodium and cause ventricular tachycardia (Decher et al., 2017b).

To conclude, the wide distribution of TREK-1 in human body, the diverse stimuli and regulations that it could respond to, and the pleiotropic roles it could play in physiology and physiopathology, make TREK-1 a promising target to explore in depth but at the same time very challenging to design modulators that are tissue-specific.

## Author Contributions

AD wrote the manuscript. MB, CH, and JM corrected it.

## Conflict of Interest Statement

The authors declare that the research was conducted in the absence of any commercial or financial relationships that could be construed as a potential conflict of interest.
